# Inoculation with *Jeotgalicoccus* sp. improves nutritional quality and biological value of Eruca sativa by enhancing amino acid and phenolic metabolism and increasing mineral uptake, unsaturated fatty acids, vitamins, and antioxidants

**DOI:** 10.3389/fpls.2024.1412426

**Published:** 2024-09-17

**Authors:** Abdelrahim H. A. Hassan, Maria Gabriela Maridueña-Zavala, Emad A. Alsherif, Abeer S. Aloufi, Shereen Magdy Korany, Mohammad Aldilami, Nahla A. Bouqellah, Ahmed M. Reyad, Hamada AbdElgawad

**Affiliations:** ^1^ School of Biotechnology, Nile University, Giza, Egypt; ^2^ Centro de Investigaciones Biotecnológicas del Ecuador (CIBE), Escuela Superior Politécnica del Litoral (ESPOL), Guayaquil, Ecuador; ^3^ Botany and Microbiology Department, Faculty of Science, Beni-Suef University, Beni-Suef, Egypt; ^4^ Department of Biology, College of Science, Princess Nourah bint Abdulrahman University, Riyadh, Saudi Arabia; ^5^ Botany and Microbiology Department, Faculty of Science, Helwan University, Cairo, Egypt; ^6^ Department of Biology, Faculty of Science, King Abdelaziz University, Jeddah, Saudi Arabia; ^7^ Department of Biology, Science College, Taibah University, Madinah, Saudi Arabia

**Keywords:** arugula, biosynthetic enzymes, antioxidant, fatty acids, flavonoids, plant growth promoting bacteria

## Abstract

Plant growth-promoting bacteria (PGPB) are considered a promising tool for triggering the synthesis of bioactive compounds in plants and to produce healthy foods. This study aimed to demonstrate the impact of PGPB on the growth, accumulation of primary and secondary metabolites, biological activities, and nutritional qualities of Eruca sativa (arugula), a key leafy vegetable worldwide. To this end, Jeotgalicoccus sp. (JW0823), was isolated and identified by using partial 16S rDNA-based identification and phylogenetic analysis. The findings revealed that JW0823 significantly boosted plant biomass production by about 45% (P<0.05) and enhanced pigment contents by 47.5% to 83.8%. JW0823-treated plants showed remarkable improvements in their proximate composition and vitamin contents, with vitamin E levels increasing by 161.5%. JW0823 induced the accumulation of bioactive metabolites including antioxidants, vitamins, unsaturated fatty acids, and essential amino acids, thereby improving the nutritional qualities of treated plants. An increase in the amounts of amino acids was recorded, with isoleucine showing the highest increase of 270.2%. This was accompanied by increased activity of the key enzymes involved in amino acid biosynthesis, including glutamine synthase, dihydrodipicolinate synthase, cystathionine γ-synthase, and phenylalanine ammonia-lyase enzymes. Consequently, the total antioxidant and antidiabetic activities of the inoculated plants were enhanced. Additionally, JW0823 improved antimicrobial activity against several pathogenic microorganisms. Overall, the JW0823 treatment is a highly promising method for enhancing the health-promoting properties and biological characteristics of E. sativa, making it a valuable tool for improving the quality of this important leafy vegetable.

## 1 Introduction

Beneficial bacteria, including endophytes and rhizobacteria, form symbiotic relationships with plants and are known as plant-growth-promoting bacteria (PGPB). PGPB stimulate plant growth and are used in agriculture to improve productivity and quality as an alternative to traditional fertilizers, promoting sustainability (Bakhshandeh et al., 2020; Saberi Riseh et al., 2021; Crecchio, 2020; Manoj et al., 2020). They maintain soil fertility (Harris, 1997), solubilize minerals like phosphate and zinc, and fix nitrogen (Pii et al., 2016; Yaghoubi Khanghahi et al., 2021b). Furthermore, they produce growth regulators (Khan, 2021), enhancing plant growth, metabolism, and stress resistance (AbdElgawad et al., 2021a, 2021b; Ghorbel et al., 2023), and improve agronomic and physiological traits in various plants (Yaghoubi Khanghahi et al., 2021a) and induce hormone production (Harris, 1997). Overall, PGPB support ecosystem survival and function (Schimel et al., 2007; Paz-Ferreiro and Fu, 2016). Among PGPB, *Jeotgalicoccus* sp. can effectively promote plant growth, colonization and show great potential in plant tolerance to abiotic stresses (Gong et al., 2016; Li et al., 2022; Misra et al., 2019; Liu et al., 2011). The genus *Jeotgalicoccus* was introduced by Yoon et al. (2003), initially encompassing two species (*J. halotolerans* and *J. psychrophilus*). This new genus was classified within the *Staphylococcaceae* family of the Firmicutes phylum. *J. huakuii* improved maize growth in alkaline soil and increased the production of bioactive compounds such as antioxidants, chlorophyll, and soluble sugars (Misra et al., 2019). Mukasheva et al. (2016) reported that *J. halotolerans* strain can produce growth-promoting hormones (IAA) and ACC deaminase enzyme, reducing ethylene levels in plants. This genus *(*ВАК1) demonstrated growth-promoting and phosphorus-solubilizing properties as well as antagonistic potential against the causative agents of fungal diseases (Mukasheva et al., 2016). *J. huakuii* NBRI 13E enhanced plant growth under salt stress (Misra et al., 2019). In this context, *J. huakuii* NBRI 13E boosted defense enzyme production and osmo-protectant (e.g., proline) accumulation, mitigating salinity stress. *J. nanhaiensis* is also known as a heavy metal tolerant bacterial strain enhancing phytoremediation potential to remediate arsenic from contaminated sites (Singh et al., 2019). Recent research has documented the heavy metal tolerance of *Jeotgalicoccus* strains (Ahamed et al., 2024; Alves et al., 2022; Kumari et al., 2022; Sharma et al., 2024). Additionally, James et al. (2024) highlighted the role of *Jeotgalicoccus-*associated plants in mitigating air pollution.

*Eruca sativa* Mill. (Arugula) is an annual herb found abundantly worldwide (Warwick, 1994) and is regarded as one of the most significant leaf vegetables, originating from the Mediterranean region (Zeven and de Wet, 1982). Valued for its health benefits, it is rich in fiber and contains antioxidants such as carotenoids, polyphenols, and vitamin C. Taramira oil, a flavorful oil, is traditionally extracted from its seeds. The plant’s aerial parts are commonly eaten raw in salads, like rocket salad. For almost two centuries, the genera *Eruca* and *Diplotaxis* have been recognized for their various health benefits and medicinal properties, including depurative, anti-inflammatory, digestive, aphrodisiac, diuretic, and rubefacient effects (Yaniv et al., 1998). Thus, enhancing the growth and tissue chemical composition of *Eruca* plants is crucial for meeting food needs. PGPB play a crucial role in this process of meeting food and population requirements.

Given the biological potential of *Jeotgalicoccus* sp. (JW0823), we hypothesized that symbiotic interactions of JW0823 with *Eruca* plants could enhance their tissues’ chemical composition, leading to improved plant growth and quality. Consequently, this study aimed to explore the effects of JW0823 on *E. sativa* by investigating its impact on plant growth, tissue chemical composition, and bioactive properties. Overall, JW0823 is introducing as a valuable tool for improving the quality of this important leafy vegetable, with the goal of improving agricultural practices and sustainability.

## 2 Materials and methods

### 2.1 Isolating, purification and identifying bacteria from the rhizosphere

Bacteria were isolated using the filtration method described by Brock (1983). Membrane filtration methods were utilized (Manaia and da Costa, 1991). The filters are placed on bacterial media plates. Bacteria were incubated on the plates for 2-7 days at 30-37°C after inverting. Bacterial biodiversity was observed, and purification of bacterial colonies was done by streaking several times on the isolation medium using the streaking plate method and then subculturing on slants of the same medium. The ability of bacteria to produce indole acetic acid (IAA) was assessed by the method described by Patten and Glick (2002), using Salkowski’s reagent [FeCl_3_ (0.5 M) solution in perchloric acid (35%)] and orthophosphoric acid.

Extracting genomic DNA and identifying PCR products were performed to determine the culture species. DNA from the isolates was extracted using the Pure Link Genomic DNA Kit (K182001), a bacterial DNA extraction kit, following the manufacturer’s procedure. The concentration of extracted DNA was also measured spectrophotometrically using a Nano Drop ND 1000 (Thermo Scientific, USA). The isolated DNA was validated using a standard agarose gel (1% w/v). PCR and Sequencing Work Purification as well as standard sequencing for PCR products were carried out by Macrogen Company (Seoul, Korea). The PCR reaction was conducted using 100 ng of genomic DNA in a total volume of 50 µl, with a reaction buffer at 1x concentration, 30 pmole of each primer, and 2 units of Taq polymerase. The thermal cycling conditions (denaturation step at 94°C for 5.5 minutes, followed by 30 cycles of denaturation at 93°C for 1 minute, primer annealing at 53°C for 1 minute, and extension at 72°C for 1.5 minutes). The PCR products were then purified using QIAquick PCR purification reagents. The gel was stained with ethidium bromide and visualized using an ultraviolet transilluminator.

Sequencing reactions were carried out in a MJ Research PTC-225 Peltier Thermal Cycler using ABI PRISM^®^ BigDyeTM Terminator Cycle Sequencing Kits with AmpliTaq^®^ DNA polymerase (FS enzyme) (Applied Biosystems), following the manufacturer’s instructions. Each template was sequenced in a single pass using the universal primer 27F (5’-AGAGTTTGATC(AC)TGCCTCAG-3’). The fluorescent-labeled fragments were isolated from unincorporated terminators using the Big Dye^®^X Terminator™ purification process. The samples were resuspended in distilled water before electrophoresis using an ABI 3730xl sequencer (Applied Biosystems). The sequences were examined for sequence similarity using BLAST (www.ncbi.nlm.nih.gov/BLAST/) (Altschul et al., 1997), and compared to reference sequences found in BLAST and downloaded from GenBank (www.ncbi.nlm.nih.gov/genbank/).

### 2.2 Experimental setup, plant materials, and growth conditions

The arugula seeds (*E. sativa*) (Agricultural Research Centre, Giza, Egypt). After that, for six hours at room temperature, sterile arugula seeds were immersed in a liquid suspension of the isolated strain, JW0823 inoculum (cultured at 30°C, pH 7.8 and 0% NaCl for 48 hours) at 25% concentrations (2.5 × 10^7^ CFU mL^−1^), while the control group was submerged in distilled water. The treated and controlled arugula seeds were sown into sterile soil and three biological replicates for each treatment are represented by the three pots. The clay soil initially contained 14.5 mg organic carbon (C), 13.7 mg nitrate-nitrogen (N), 1.7 mg ammonium-N, 9.3 mg phosphorus (P)/g air dry soil at a humidity of 0.41 g water/g dry soil. The soil was watered twice a day and maintained at 58%. The arugula was cultivated in pots (20 cm high and 15 cm width) and grown in growth-controlled chambers with the following conditions: 24°C, 290 ± 12 µmol PAR m^−2^ s^−1^, 16 hours of light and 8 hours of darkness, and 58% relative humidity. All pots were arranged in a randomized complete block design with five replicates per treatment. After 5 weeks of growth, the fresh weight (FW) and dry weight (DW) of the shoots were measured and kept at -80°C pending biochemical studies. Ultimately, the arugula plants were preserved for additional examination by freezing them in liquid nitrogen at -196°C.

### 2.3 Determination of photosynthetic rate

An EGM-4 infrared gas analyzer connected to an Environmental Monitor Sensor Probe Type 3 (PP Systems, Hitchin, UK) was used to determine the photosynthetic rate (Lichtenthaler, 1987).

A net CO_2_ exchange (NE) measurement was conducted under ambient light, followed by a dark respiration measurement with the enclosure covered with a dark cloth for180 s measurement.

### 2.4 Pigment analysis

A MagNALyser (Roche, Vilvoorde, Belgium) was employed to homogenize 200 mg of plant in acetone at 7000 rpm for one minute. Subsequently, they underwent centrifugation for 20 minutes at 14,000 × g and 4°C. Following the method described by Almuhayawi et al. (2020), the supernatant was filtered and subjected to HPLC analysis using a Shimadzu SIL10-ADvp system equipped with a reversed-phase column at 4°C. Carotenoids were isolated using a silica-based C18 column with acetonitrile/methanol/water (81:9:10) and methanol/ethyl acetate (68:32) as the solvents. A diode-array detector (Shimadzu SPDM10Avp) was employed to extract and identify β-carotene, chlorophyll a, and chlorophyll b at wavelengths of 420, 440, 462, and 660 nm.

### 2.5 Determination of the nutritional quality

Data about the proximate composition, amounts of amino, organic, and fatty acids, as well as minerals, vitamins, and phenolics were obtained according to the following methods, in order to provide insight into the nutritional quality of ***E. sativa*** plants.

#### 2.5.1 Proximate composition analysis

Following Wong et al. (2000)’s procedure, the carbohydrate content of each arugula plant group, whether treated or untreated with PGPB, was determined. Additionally, protein concentration (0.2 g FW) in each plant sample was extracted in 0.1 mM KPO_4_ buffer at pH 7. Then it was measured according to the method described by Lowry et al. (1951). After that, it was measured using the Lowry et al. (1951) method. The total lipid content of the plants was assessed after homogenizing the samples in a 1:2 (v/v) mixture of methanol and chloroform, as outlined by Shiva et al. (2018). Subsequently, the plants were centrifuged at 3000× g for 15 minutes, and the resulting pellets were dissolved in a 4:1 (v/v) mixture of ethanol and toluene. To determine the total lipid content, the lipids were first concentrated and then quantified using a gravimetric method. The results were expressed as milligrams of lipid per gram of fresh plant weight. The crude fibers were isolated from the plant material (Lee, 2002). The enzymatic digestion was performed using protease at pH 7.6 and 55°C for 24 minutes, followed by treatment with amyloglucosidase at pH 6 and 0°C for 30 minutes to eliminate proteins and starches.

#### 2.5.2 Elemental analysis

Following the methodology outlined by AbdElgawad et al. (2014), 200 mg of plants, treated with bacterial endophytic inoculation and control plants, were subjected to digestion in a 5:1 (v:v) HNO_3_/H_2_O solution to determine their mineral composition. Subsequently, both major and trace elements were analyzed using inductively coupled plasma mass spectrometry (ICP-MS) on a Finnigan Element XR instrument from Scientific, Bremen, Germany.

#### 2.5.3 Amino acids metabolism

Following the protocol outlined in Sinha et al. (2013), 100 mg of every plant sample were dissolved in five mL ethanol (80%), while being spun at 5000 rpm for one minute. Subsequently, a 25-minute centrifugation at 14,000 x g was conducted, and the supernatant that resulted was reconstituted in 5 milliliters of chloroform. Then, one milliliter of water was used to remove any residue. After resuspension in chloroform, the pellet and supernatant were centrifuged for 10 minutes at 8000× g. Then filtration through Millipore microfilters with 0.2-μm pore size was done. Elution (A, containing 10% acetonitrile, 84% ammonium formate and 6% formic acid, v/v) and quantification of amino acids (B, containing 2% formic acid, v/v and acetonitrile) were carried out using a Waters Acquity UPLC TQD apparatus connected to a BEH amide column. A set of amino acid standards was utilized as the reference.

Glutamyl synthase (GS) activity was measured using the methodology described by Almuhayawi et al. (2021), with extraction conducted in 100 mg mL^−1^ Tris-HCl (50 mM), pH 7.4, containing 2% polyvinylpyrrolidone, 4 mM DTT, 10 mM MgCl_2_, 1 mM EDTA, 10% glycerol, and 2 mM PMSF. Subsequently, γ-glutamyl hydroxamate synthesis was assessed, indicating the presence of GS activity in a Tris-acetate reaction buffer (200 mM, pH 6.4). Dihydrodipicolinate synthase (DHDPS) activity was carried out as per Kumpaisal et al. (1987). Plants not exposed to L-aspartate-b-semialdehyde were used as a negative control. The reaction was conducted at 36.5°C to facilitate adduct formation between the reaction product and o-ABA. Trichloroacetic acid (TCA) at a concentration of 12% was added to stop the process, and samples were analyzed at 550 nm following a 60-minute dark incubation period.

Cystathionine γ-synthase (CGS) was extracted in 20 mM MOPS for 15 minutes at 4°C. The supernatants were combined with a reaction buffer containing O-phospho-homoserine (5 mM), L-cysteine (2 mM), PLP (100 µM), and AVG (200 µM). L-cystathionine formation was isolated using a phenomenex Hyperclone C18 BDS column on a Dionex HPLC system, following the method described by Ravanel et al. (1998).

#### 2.5.4 Organic acid analysis

Organic acids in 200 mg of plant samples were detected using HPLC (0.001 N sulfuric acid, at 210 nm, flow rate of 0.6 mL min^−1^), following the method outlined by Hamad et al. (2015). The detection system comprised an LED model detector (Ultimate 3000) and a liquid chromatographer (Dionex, Sunnyvale, CA, USA), equipped with an LPG-3400A pump, a TCC-3000SD column thermostat, and an EWPS-3000SI autosampler. The separation was conducted at 65°C using an Aminex HPH-87 H (300 × 7.8 mm) column with an IG Cation H (30 × 4.6) precolumn from Bio-Red company. UV detection system operating at 210 nm was used to estimate the concentrations of citric, succinic, fumaric, and malic acids (LaChromL-7455 diode array, LaChrom, Tokyo, Japan). Data analysis was performed using the Chromeleon v.6.8 computer program.

#### 2.5.5 Fatty acid analysis

200 mg of the plant samples were extracted in 50% aqueous methanol at 25°C. The plant material was thoroughly homogenized in 50% aqueous methanol to ensure efficient extraction. The mixture was then subjected to shaking, and then the extract was centrifuged at 4°C for 20 minutes. The GC/MS (Hewlett Packard, Palo Alto, CA, USA) was used to sperate and identify fatty acid detection method, which was equipped with an HP-5 MS column (30 m × 0.25 mm × 0.25 mm). NIST 05 database and Golm Metabolome (http://gmd.mpimp-golm.mpg.de, accessed on February 23, 2024) were utilized. A set of amino acid standards was utilized as the reference.

#### 2.5.6 Vitamin content analysis

To determine the levels of vitamins in plant samples, approximately 200 mg of fresh plant material was analyzed using UV and/or fluorescence detectors. For this analysis, thiamine and riboflavin contents were measured. A reverse-phase C18 column was utilized for the separation process in high-performance liquid chromatography (HPLC), with a methanol/water solvent system. Vitamin C (ascorbate) levels were quantified using HPLC with Shimadzu equipment (Hertogenbosch, The Netherlands). Antioxidants were isolated from plant tissues that had been extracted in 1 mL of ice-cold 6% (w/v) meta-phosphoric acid. The antioxidants were subsequently separated on a reversed-phase HPLC column (Farfan-Vignolo and Asard, 2012). The thiamine and riboflavin contents were again determined using UV and/or fluorescence detection, with a reverse-phase C18 column used for separation.

#### 2.5.7 Analysis of phenolics and their biosynthetic enzymes

To assess the total flavonoid and phenolic concentrations, plant material (120 mg) was homogenized in 80% ethanol. After centrifugation at 4°C for 20 minutes, the phenolic content was determined using the Folin–Ciocalteu reagent, with gallic acid as the standard reference. Flavonoid content was measured using a modified colorimetric method with aluminum chloride, with quercetin serving as the calibration standard. For evaluating phenylalanine ammonia-lyase (PAL) activity, 0.25 g of frozen plant material was homogenized in a Tris-HCl buffer (100 mM, pH 8.8) containing L-phenylalanine (40 mM). The enzyme activity was assessed by measuring the absorbance of transcinnamic acid produced at 290 nm, following the protocol by AbdElgawad et al. (2014). Water was used as a negative control in place of the plant samples to ensure accuracy in the enzyme assay.

### 2.6 Biological activities

#### 2.6.1 Antioxidant activities

Several tests were used to assess the plants’ antioxidant potential (Almuhayawi et al., 2020). About 0.1 g was extracted in 80% ethanol to determine the ferric reducing antioxidant power (FRAP). Centrifugation was then carried out (14,000 rpm, 20 min). Next, 0.1 mL of the extract was combined with 20 mM FeCl_3_ in 0.25 M acetate buffer, which is known as the FRAP reagent. 2.4 mM potassium persulphate was combined with 2,20-azino-bis (3-ethylbenzothiazoline-6-sulfonic acid) (ABTS) to determine its concentration. The absorbance was measured at 734 nm, and 0.1 mL of the extract and 0.25 mL of the DPPH reagent were used to detect the DPPH activity. At 517 nm, the absorption was found.

#### 2.6.2 Antimicrobial activity

##### 2.6.2.1 Assessment of antibacterial activity in plants

The antibacterial activity of the plant extracts was evaluated using the standard dilution method as outlined by Almuhayawi et al. (2021). Initially, 100 mg of the plant material was extracted in dimethyl sulfoxide (DMSO). The resulting extract was then used to prepare the test medium, which was supplemented with 0.1 mL of a 1:10,000 dilution of a liquid culture of the reference strain *Staphylococcus aureus* ATCC 6538 P. This dilution contained approximately 10^4^–10^5^ bacterial cells per mL. The inoculated media was incubated at 37°C for 18 hours.

To determine the antimicrobial efficacy of the plant extracts, the Minimum Inhibitory Concentration (MIC) was measured. The extracts were tested against a range of bacterial and fungal species, including *Candida glabrata* (ATCC 90030), *Pseudomonas aeruginosa* (ATCC 10145), *Enterobacter aerogenes* (ATCC 13048), *Proteus vulgaris* (ATCC 8427), *Staphylococcus saprophyticus* (ATCC 19701), *Escherichia coli* (ATCC 29998), *Salmonella typhimurium* (ATCC 14028), *Staphylococcus epidermidis* (ATCC 12228), *Candida albicans* (ATCC 90028), *Streptococcus salivarius* (ATCC 25975), *Aspergillus flavus* (ATCC 9170), *Enterococcus faecalis* (ATCC 10541), and *Serratia marcescens* (ATCC 99006).

For comparative purposes, ciprofloxacin (25 mg/mL) was used as a positive control, while 100% DMSO served as a negative control. This setup allowed for assessment of the antibacterial effectiveness of the plant extracts against various pathogens.

#### 2.6.3 Antidiabetic activity

##### 2.6.3.1 Inhibition of α-amylase assay

The α-amylase inhibitory activity was assessed following a modified method from Dada et al. (2017). To begin, 500 µL of the plant extract was mixed with 500 µL of 0.02 M sodium phosphate buffer (pH 6.9, containing 0.006 M NaCl) and 1.0 U/mL of α-amylase solution. This mixture was incubated at 25°C for 10 minutes. Following pre-incubation, 500 µL of 1% starch solution, also prepared in 0.02 M sodium phosphate buffer (pH 6.9 with 0.006 M NaCl), was added to the mixture. The reaction was allowed to proceed at 25°C for another 10 minutes. To halt the reaction, 1.0 mL of DNS (dinitrosalicylic acid) color reagent was added. The test tubes were then heated in boiling water for five minutes, cooled to room temperature, and subsequently diluted with 10 mL of distilled water. The absorbance of the final solution was measured at 540 nm. The inhibitory activity of the plant extract against α-amylase was determined by comparing its effect to that of a control.

##### 2.6.3.2 α-glucosidase inhibition assay

For the α-glucosidase inhibition test, a modified version of the protocol described by Dada et al. (2017) was used. Plant extract (500 µL) was diluted with 100 µL of 0.1 M potassium phosphate buffer (pH 6.9) containing 1.0 U/mL of α-glucosidase solution and incubated at 25°C for 10 minutes in a 96-well plate. After the pre-incubation, 50 µL of a 5 mM solution of p-nitrophenyl-α-D-glucopyranoside in 0.1 M potassium phosphate buffer (pH 6.9) was added to each well. The reaction was conducted at 25°C for five minutes. The inhibition % of α-glucosidase activity was calculated for the plant extract and compared to the control.

##### 2.6.3.3 Estimation of glycemic index

The glycemic index of the plant extract was determined following the procedure outlined by Brouns et al. (2005). For this analysis, the samples were extracted using 80% ethanol, and the glycemic index was evaluated according to the specified method.

### 2.7 Statistical analyses

The SPSS program was employed to determine the statistical analyses (SPSS Inc., Chicago, IL, USA). A T-test was performed to determine the differences between means. Each experiment was conducted in triplicate (n = 3). All parameters were subjected to cluster analysis using the MultiExperiment Viewer (MeV) TM4 software (Dana-Farber Cancer Institute, Boston, MA, USA), based on Pearson’s distance metric.

## 3 Results

### 3.1 Bacterial identification and its characterizations

**Figure 1:**
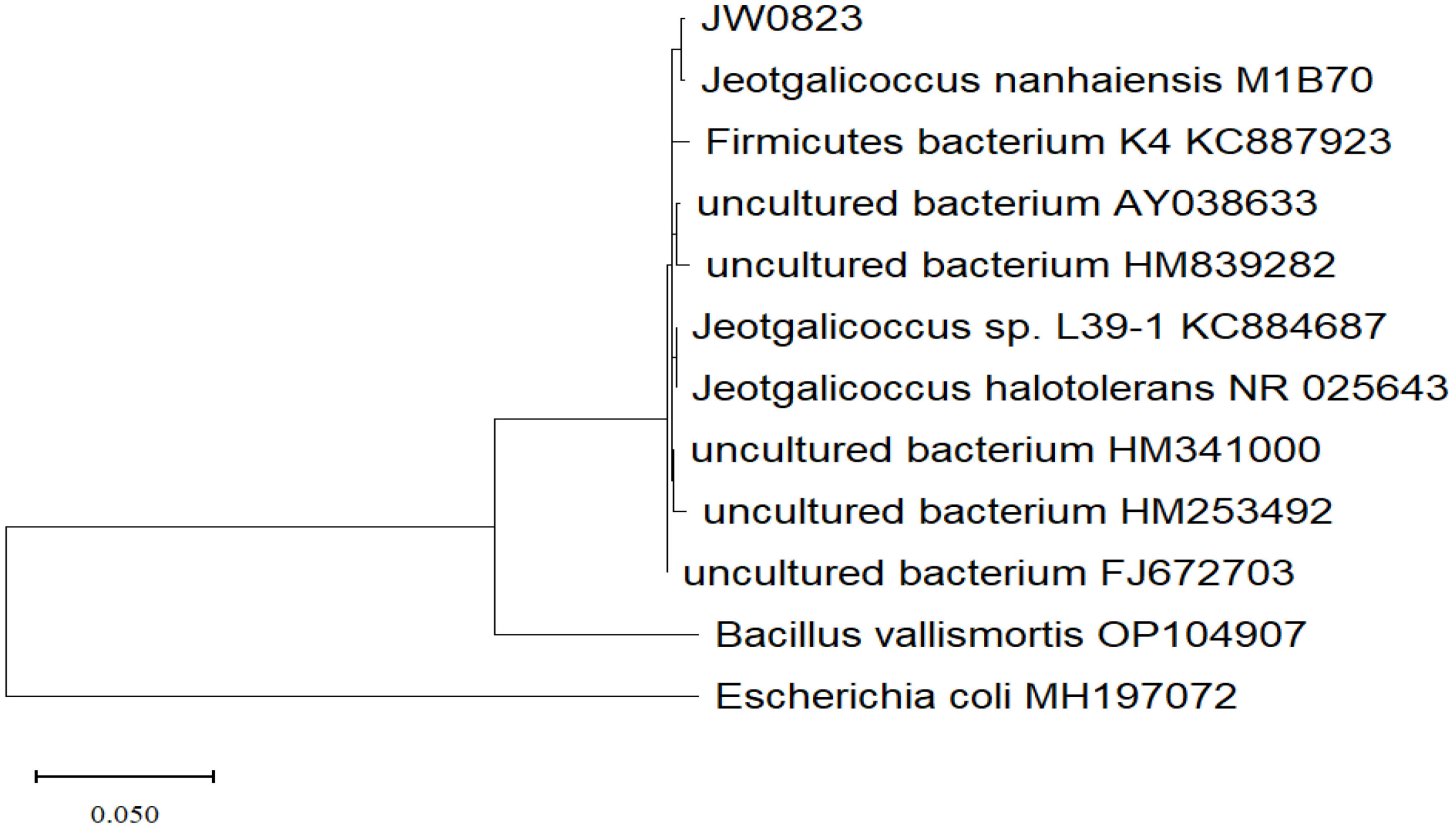
Phylogenetic tree based on 16S rRNA gene sequences, constructed by the Maximum of likelihood method using MegaX application, showing the position of strain JW0823 among closest species available at GenBank.

The isolate was identified using biochemical and molecular methods, while 16S rDNA sequencing analysis revealed that strain JW0823 is 96.13% linked to the genus *Jeotgalicoccus*. The biochemical analysis demonstrated that the bacterial isolate JW0823 decomposed tyrosine and produced acid from arabinose, D-mannitol, and sucrose. The isolate has a broad growth range. It showed growth at pH levels ranging from 5 to 9, with an optimal pH of 8. The isolate could grow at 0% and 15% NaCl, with an optimal NaCl of 7-8%, and no growth above 20% NaCl. Furthermore, the isolate exhibited a growth temperature range of 15-55°C, with an optimal temperature of 28-40°C. The isolated JW0823 can produce IAA (**Table 1**). The phylogenetic tree (**Figure 1**) showed that strain MD36 is closely related to JW0823.

**Table 1:** Biochemical activities of JW0823 bacteria.

Biochemical activities	JW0823 bacteria
Nitrate reduction	–
Decomposition of:	
Hypoxanthine Starch Tween 80 Tyrosine Xanthine	- - - + -
Acid production from:	
Arabinose Fructose D-Galactose D-Glucose Glycerol Maltose D-Mannitol Sucrose	+ - - - - - + +
Growth at:	
pH Range Optimum	5-9 8
0% NaCl 15% NaCl 20% NaCl	+ + -
Temperature (°C): Range Optimum	15-55 28-40

### 3.2 Bacterial colonization improved the biomass and photosynthesis of *E. sativa*

**Figure 2:**
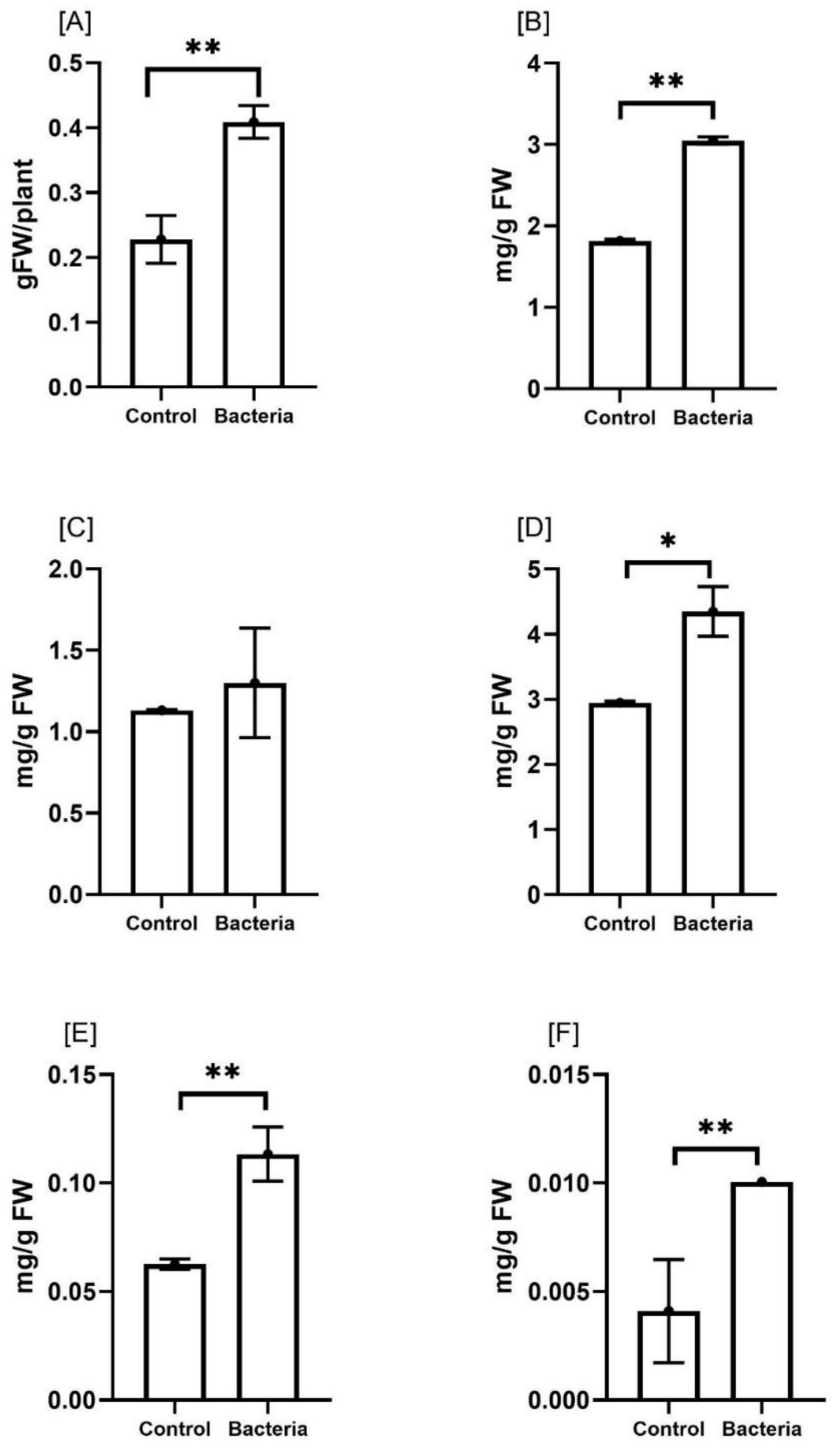
Effect of JW0823 bacteria on the biomass and pigment contents of *Eruca sativa* represented by **(A)** the biomass **(B)** chlorophyll a, **(C)** chlorophyll b, **(D)** chlorophyll a+b, **(E)** Beta carotene, and **(F)** lycopene. Data are represented by means ± standard errors. Bars flagged with 1, or 2 asterisks indicate significant differences between JW0823 bacteria-treated and control groups at p<0.05, or p<0.01 respectively.

The effect of JW0823 colonization on the biomass, photosynthesis and pigment contents of *E. sativa* was studied (**Figure 2**). JW0823 -inoculated plants showed a significant increase in biomass production by about 78.2% in comparison with untreated control plants (*p*<0.05). Generally, plant growth is closely related to the efficiency of photosynthesis, so the pigment contents were estimated. It was noticeable that chlorophyll a, chlorophyll b, and chlorophyll a+b contents were enhanced in the treated plants by about 67.5%, 15%, and 47.4% respectively, and these elevations were significant (*p* < 0.05) in the case of chlorophyll a and chlorophyll a+b only. In addition, β-carotene and lycopene pigments exhibited significant increases of about 83.3% and 150% respectively (*p* < 0.01).

### 3.2 JW0823 induced the proximate composition, minerals, and vitamins of *E. sativa*

**Figure 3:**
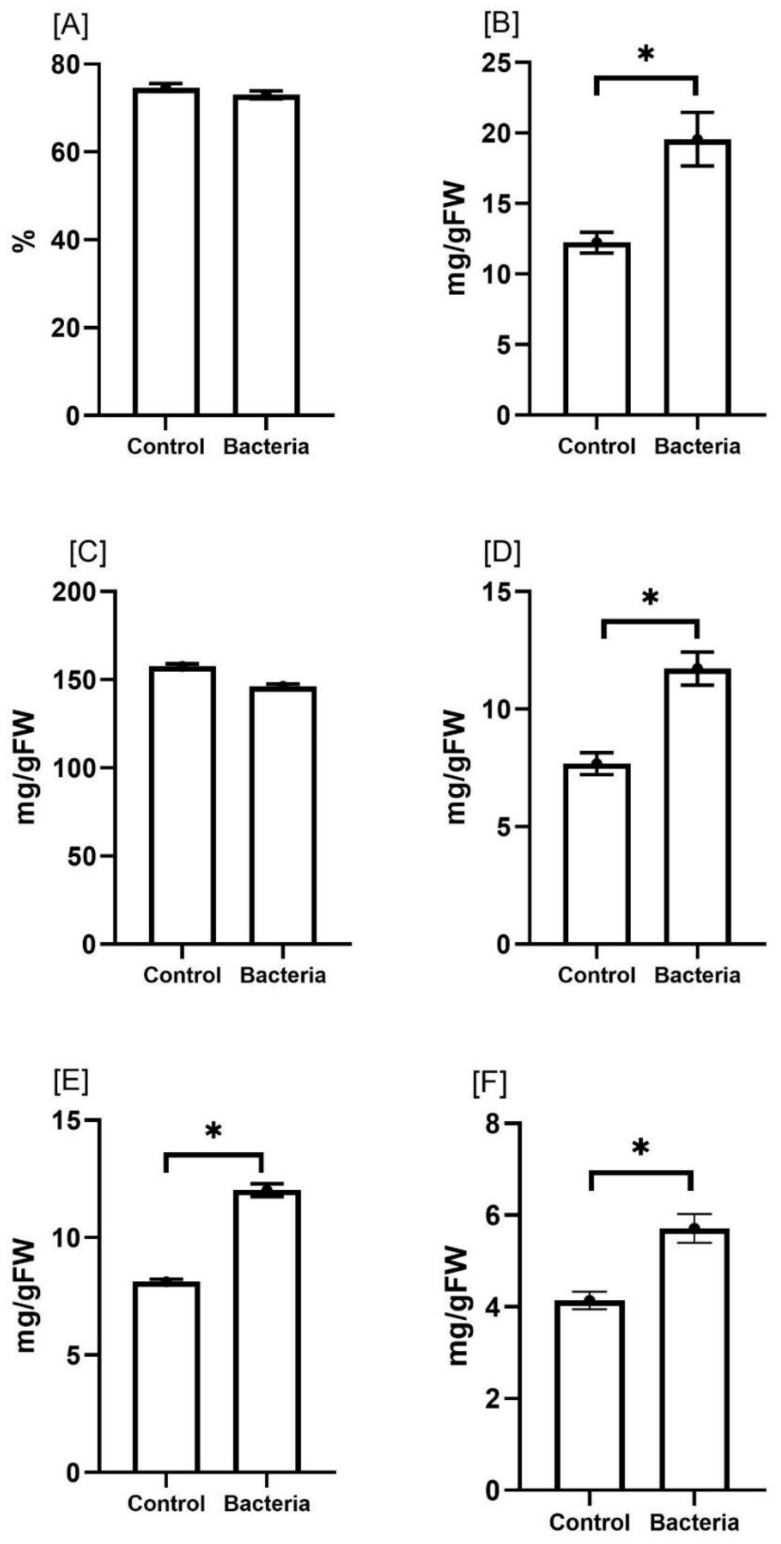
Effect of JW0823 bacteria on the proximate composition of *Eruca sativa* represented by **(A)** moisture, **(B)** total proteins, **(C)** total lipids, **(D)** crude fibers, **(E)** carbohydrates and **(F)** ash. Data are represented by means ± standard errors. Bars flagged with an asterisk indicate significant differences between JW0823 bacteria-treated and control groups at p<0.05.

To assess the effect of mycorrhizal colonization on the proximate composition of *E. sativa*, we determined total protein, fat, crude fiber, ash, and carbohydrate contents in treated versus untreated plants (**Figure 3**). The proximate composition of the JW0823-treated plants showed significantly higher contents of total protein, crude fiber, ash, and carbohydrate by about 59.9%, 52.7%, 37.9% and 48%, respectively, compared to control samples (*p* < 0.05). On the other hand, moisture and fat percentages showed a slight decline in treated plants compared to untreated ones. Concerning mineral contents (**Table 2**), bacterial-treated plants displayed significantly higher contents (*p* < 0.05) in all measured minerals except Cu, Fe, and Mn (**Table 3**). The highest increases were recorded for K and Na by 119.1% and 100%, respectively. Both Zn and Mn contents exhibited an increase of 50% in treated plants compared to the untreated plants. About a 59.8% increase in Mg and 45.9% in phosphorus content was observed, whereas only a 5% increase in Fe content was recorded by the inoculated plants.

**Table 2:** Effect of JW0823 bacteria on the levels of minerals in *Eruca sativa*.

Minerals (mg/g)	Control	JW0823 bacteria
**K**	11.474 ± 0.047	25.228 ± 0.55*
**Na**	1.561 ± 0.021	3.213 ± 0.02*
**Ca**	1.485 ± 0.177	1.872 ± 0.258*
**Cu**	0.008 ± 0	0.009 ± 0.002*
**Fe**	0.18 ± 0.015	0.172 ± 0.029
**P**	1.108 ± 0.088	1.625 ± 0.02*
**Zn**	0.1 ± 0.007	0.147 ± 0.006*
**Mn**	0.024 ± 0.001	0.034 ± 0
**Mg**	2.588 ± 0.263	4.143 ± 0.385*

Data are represented by means ± standard errors. Means with an asterisk indicate significant differences between treated and control groups at p<0.05.

**Table 3:** Effect of JW0823 bacteria on amino acid contents in *Eruca sativa*.

Amino acids (mg/g)	Control	JW0823 bacteria
**Aspartic acid**	13.55 ± 0.5	6.54 ± 1.38*
**Glutamic acid**	12.75 ± 0.81	16.57 ± 0.41*
**Glutamine**	11.79 ± 0.6	14.53 ± 0.46*
**Serine**	6.01 ± 0.09	8.78 ± 0.86*
**Glycine**	11.44 ± 1.08	8.01 ± 0.23*
**Arginine**	21.68 ± 3.4	31.61 ± 9.47*
**Alanine**	3.49 ± 0.18	2.97 ± 0.22
**Histidine**	5.79 ± 0.15	2.03 ± 1.33*
**Valine**	9.07 ± 0.21	2.09 ± 1.47*
**Methionine**	1.14 ± 0.48	2.89 ± 0.04*
**Cystine**	1.53 ± 0.02	1.59 ± 0.15
**Isoleucine**	2.42 ± 1.08	8.96 ± 2.16*
**Leucine**	14.78 ± 1.05	10.27 ± 1.39*
**Tyrosine**	6.48 ± 0.15	4.69 ± 0.33*
**Lysine**	17.32 ± 1.23	14.28 ± 0.15*
**Threonine**	6.58 ± 0.57	3.01 ± 0.33*
**Tryptophan**	0.55 ± 0.02	0.39 ± 0
**Glutamine synthase**	4.91 ± 0.28	6.22 ± 0.01
**Dihydrodipicolinate synthase (DHDPS) (lysine biosynthase)**	1.94 ± 0.11	3.29 ± 0.1*
**Cystathionine γ-synthase (CGS) (methionine biosynthase)**	0.01 ± 0	0.03 ± 0
**Phenylalanine**	22.87 ± 2.47	33.87 ± 3.77*
**Phenylalanine aminolyase**	4.2 ± 0.37	7.01 ± 0.92*

Data are represented by means ± standard errors. Means with an asterisk indicate significant differences between treated and control groups at p<0.05.

**Figure 4:**
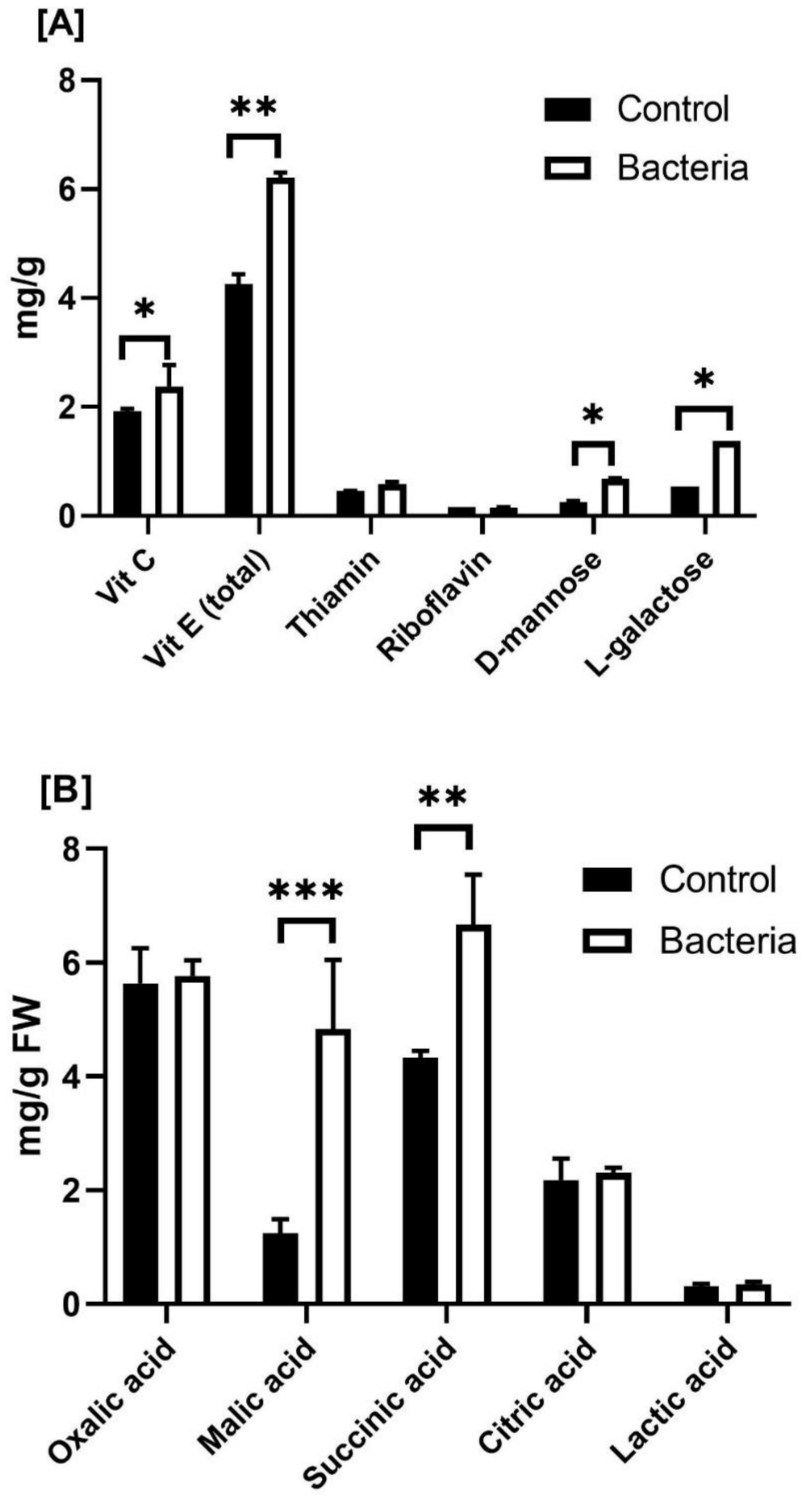
Effect of JW0823 bacteria on the levels of **(A)** vitamins and **(B)** organic acids in *Eruca sativa*. Data are represented by means ± standard errors. Bars flagged with 1, 2, or 3 asterisks indicate significant differences between JW0823 bacteria-treated and control groups at p<0.05, p<0.01, or p<0.001, respectively.

Regarding the impact of JW0823 on vitamin contents, the treated plants exhibited significantly higher levels of each examined vitamin (**Figure 4A**). Inoculated plants exhibited significantly higher levels of vitamin C, vitamin E, D-mannose, and L-galactose than untreated plants, with increases of approximately 23.9%, 46.11%, 161.5%, and 140.7%, respectively (*p* < 0.05).

### 3.3 JW0823 enhanced the functional food potentiality of *E. sativa*

To investigate the impact of JW0823 treatment on the functional food potential, nutritional quality, and bioactive metabolites of *E. sativa*, we measured the levels of amino acids (**Table 3**), fatty acids (**Table 4**), organic acids (**Figure 4B**), and phenolics (**Table 5**) in treated and untreated plants. Nine out of twenty-two examined amino acids in the treated plants were significantly increased when inoculated with JW0823 compared to control ones (p<0.05). However, some amino acids decreased by JW0823 treatment, such as aspartic acid, glycine, alanine, histidine, valine, and threonine. Isoleucine, cystathionine γ-synthase (CGS), and methionine showed the highest increments of 270.2%, 200%, and 153.5%, respectively (*p*<0.05).

**Table 4:** Effect of JW0823 bacteria on fatty acids (%) in *Eruca sativa*.

Fatty acids (%)	Control	JW0823 bacteria
Tetradecanoic (C14:0)	1.29 ± 0.14	0.78 ± 0.2
Pentadecanoic (C16:0)	13.7 ± 2.53	16.87 ± 1.71*
Eicosanoic (C20:0)	1.93 ± 0.1	1.81 ± 0.33
Docosanoic (C22:0)	1.66 ± 0.25	1.43 ± 0.06
Octadecanoic (C18:0)	7.98 ± 0.68	11.45 ± 1.83*
Pentacosanoic (C24:0)	0.08 ± 0.03	0.15 ± 0.03
Total saturated fatty acids	26.64 ± 2.49	32.48 ± 2.34*
Pentadecanoic (C16:1)	2.26 ± 0.23	2.98 ± 0.62
Pentadecanoic (C16:1)	0.6 ± 0.15	1.29 ± 0.16*
Pentadecanoic (C16:3)	2.45 ± 0.27	1.36 ± 0.11*
Octadecanoic (C18:1)	3.85 ± 0.59	5.95 ± 0.46*
Octadecanoic (C18:2)	31.67 ± 6.57	23.82 ± 0.81*
Heptadecanoic (C18:3)	34.75 ± 8.76	34.4 ± 7.56
Heptadecanoic (C18:4)	2 ± 0.46	1.29 ± 0.16*
Tetracosanoic (C20:3)	0.45 ± 0.18	0.36 ± 0.11
Total fatty acids	78.03 ± 15.49	71.47 ± 7

Data are represented by means ± standard errors. Means with an asterisk indicate significant differences between treated and control groups at p<0.05.

**Table 5:** Effect of JW0823 bacteria on the concentrations of phenolics in *Eruca sativa*.

Phenolics (mg/g FW)	Control	JW0823 bacteria
Total Phenolics	569.536 ± 5.283	1065.631 ± 29.584*
Total Flavonoids	60.662 ± 3.444	116.995 ± 2.954*
Caffeic acid	0.015 ± 0.001	0.026 ± 0
Ferulic acid	1.583 ± 0.025	2.429 ± 0.033*
Protocatechuic acid	0.384 ± 0.137	0.327 ± 0.003
Catechin	0.473 ± 0.019	0.77 ± 0.019
Galic acid	11.959 ± 0.537	18.911 ± 0.432*
p-Coumaric acid	2.244 ± 0.142	4.249 ± 0.072*
Cinnamic acid	2.564 ± 0.123	4.18 ± 0.088*
Resorcinol	0.052 ± 0.008	0.071 ± 0.001
Chlorogenic acid	0.153 ± 0.007	0.241 ± 0.005*
Syringic acid	0.693 ± 0.016	1.523 ± 0.033*
Quercetin	1.406 ± 0.132	2.499 ± 0.052*
Quercetrin	0.135 ± 0	0.268 ± 0.011*
Luteolin	0.046 ± 0.004	0.102 ± 0.003*
Apigenin	0.293 ± 0.03	0.577 ± 0.018*
Isoquercetrin	0.63 ± 0.085	1.264 ± 0.022*
Rutin	1.005 ± 0.102	2.008 ± 0.059*
Ellagic acid	0.258 ± 0.023	0.403 ± 0.009*
Velutin	0.272 ± 0.013	0.454 ± 0.008*
Naringenin	0.004 ± 0	0.007 ± 0
Genistein	0.002 ± 0	0.003 ± 0
Daidzein	0.001 ± 0	0.003 ± 0
Fisetin	0.002 ± 0	0.003 ± 0
O-hydroxydaidzein	0.002 ± 0	0.003 ± 0

Data are represented by means ± standard errors. Means with an asterisk indicate significant differences between treated and control groups at p<0.05.

The five estimated organic acids recorded increases in the treated plants, with malic acid exhibiting the highest increase of 287.2% and oxalic acid the lowest increase of 2.3% (**Figure 4B**). Similarly, five out of sixteen detected fatty acids in the target plant were significantly increased when inoculated with JW0823 compared to control plants, whereas some fatty acids showed a decrease. Pentacosanoic acid (C24:0), octadecanoic acid (C18:1), and octadecanoic acid (C18:0) recorded increases in treated plants by 87.5%, 54.5%, and 43.4%, respectively (*p*<0.05).

Additionally, all detected phenolic metabolites exhibited significant increases in JW0823-treated plants except protocatechuic acid, which showed a decrease of 14.8%. Genistein and luteolin recorded the highest increases by 50% and 121.7%, respectively, followed by fisetin and o-hydroxydaidzein, which exhibited an increase of 50% each in the inoculated plants compared to untreated plants (*p*<0.05).

### 3.4 JW0823 boosted the biological activities of *E. sativa*

#### 3.4.1 Antioxidant activity

**Figure 5:**
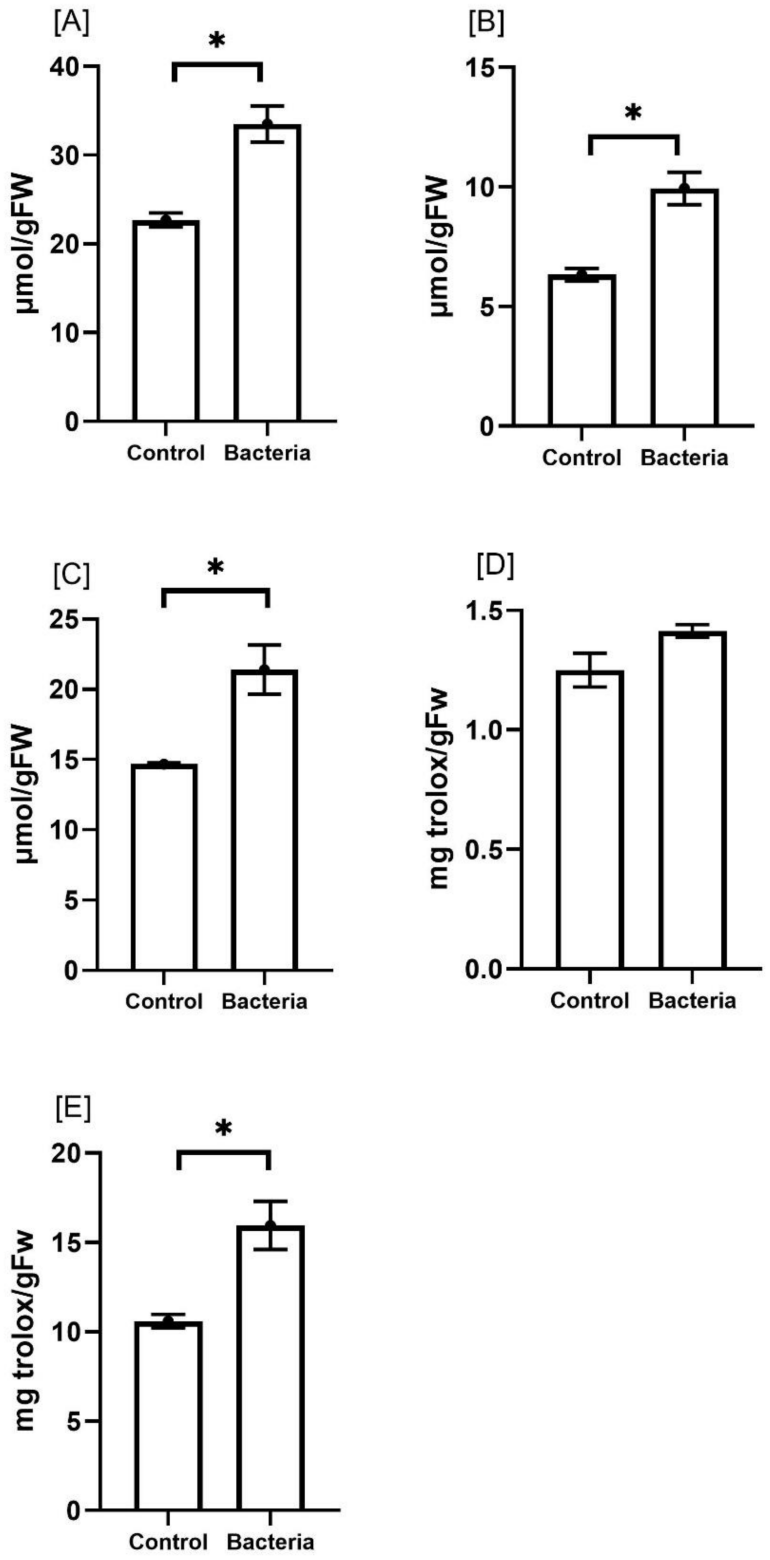
Effect of JW0823 bacteria on the total antioxidant activities of *Eruca sativa*: **(A)** total phenolics, **(B)** total flavonoids, **(C)** FRAP, **(D)** ABTS and **(E)** DPPH. Data are represented by means ± standard errors. Bars flagged with an asterisk indicate significant differences between JW0823 bacteria-treated and control groups at p<0.05.

To evaluate how JW0823 treatment affects the overall antioxidant activity of *E. sativa*, we analyzed the levels of total phenolics, total flavonoids, FRAP, ABTS, and DPPH in both treated and untreated plants (**Figure 5**). The plants treated with JW0823 exhibited significant increases in antioxidant activity, with total phenolics, total flavonoids, FRAP, ABTS, and DPPH levels increasing by approximately 48.2%, 56.9%, 46.5%, 13.2%, and 50.5%, respectively, compared to the untreated controls (*p*<0.05). These improvements in antioxidant activity were associated with higher phenolic content increased by JW0823 inoculation.

#### 3.4.2 Antimicrobial activity

**Figure 6:**
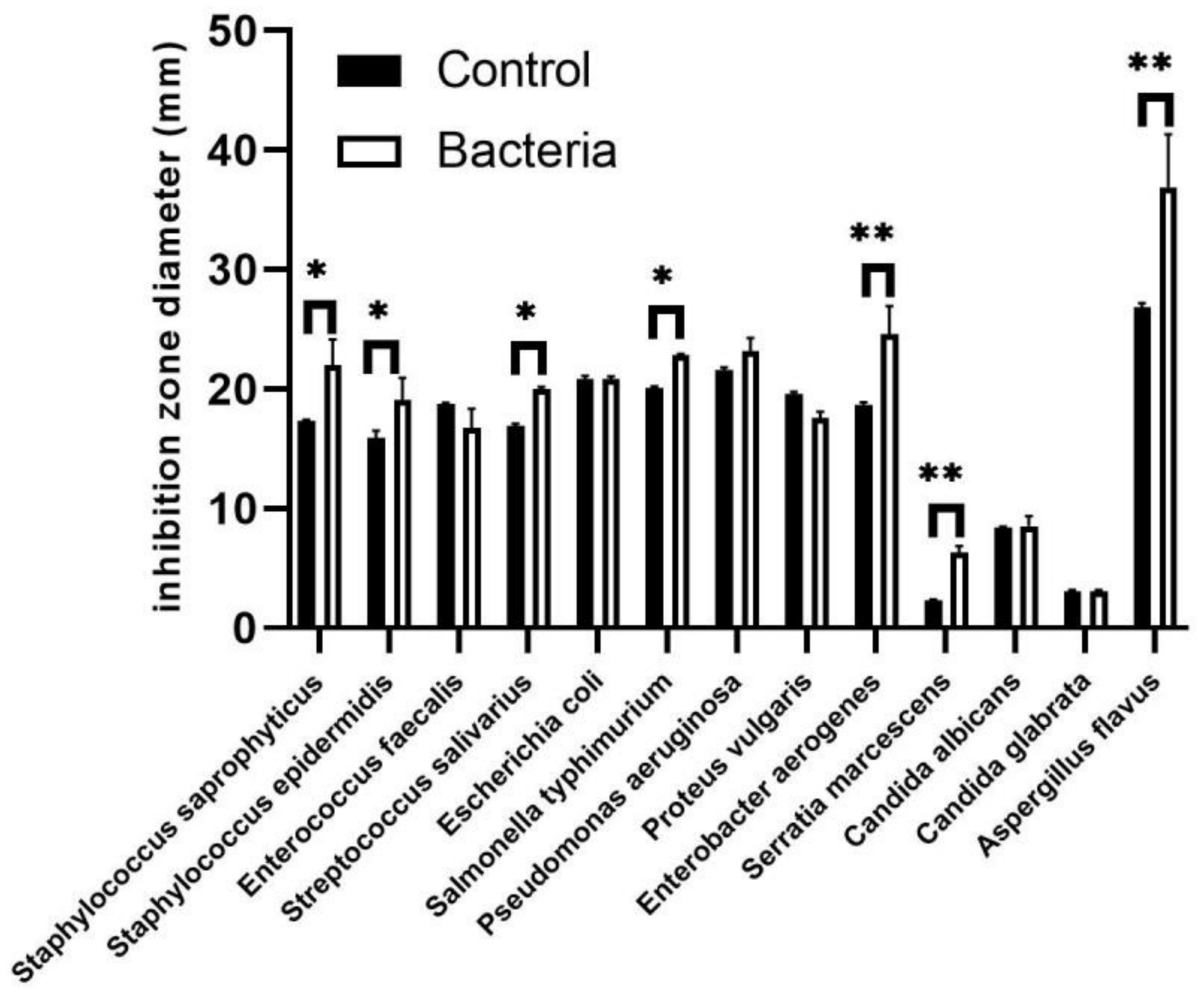
Effect of JW0823 bacteria on the antimicrobial activities of *Eruca sativa*. Data are represented by means ± standard errors. Bars flagged with 1 or 2 asterisks indicate significant differences between JW0823 bacteria-treated and control groups at p<0.05 or p<0.01, respectively.

Furthermore, both inoculated and non-inoculated plant extracts demonstrated antimicrobial activity against various bacterial and fungal species. The most pronounced effects were observed in extracts from inoculated plants, particularly against *Aspergillus flavus*, *Enterobacter aerogenes*, and *Pseudomonas aeruginosa*, as indicated by the inhibition zone diameters (**Figure 6**). The results showed that JW0823 inoculation led to significant enhancements in antimicrobial activity of *E. sativa* against *Staphylococcus saprophyticus, Staphylococcus epidermidis, Streptococcus salivarius, Salmonella typhimurium, Enterobacter aerogenes, Serratia marcescens*, and *Aspergillus flavus*, with increases of approximately 27.1%, 19.9%, 18.1%, 13.5%, 31.7%, 165%, and 37.3%, respectively (*p*<0.05) (**Figure 6**).

#### 3.4.3 Antidiabetic activity

**Figure 7:**
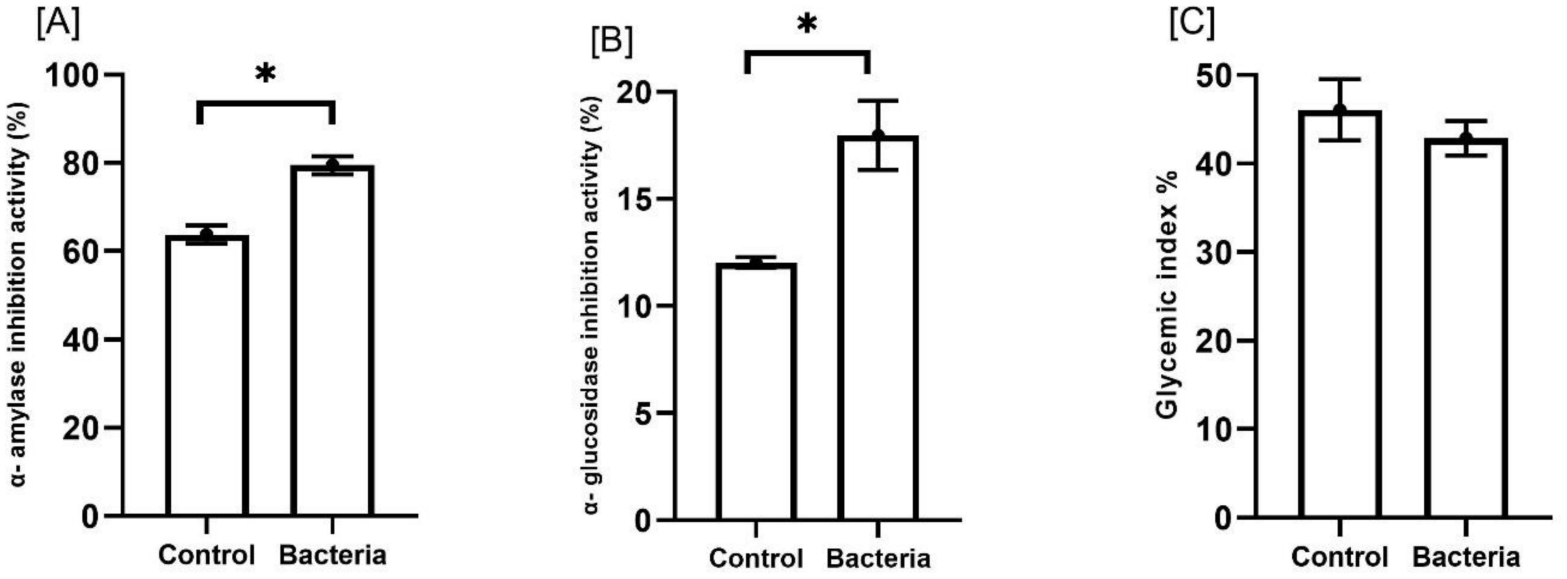
Effect of JW0823 bacteria on the antidiabetic activities of *Eruca sativa* sprouts represented by **(A)** α- amylase inhibition activity (%), **(B)** α- glucosidase inhibition activity (%), and **(C)** glycemic index %. Data are represented by means ± standard errors. Bars flagged with an asterisk indicate significant differences between JW0823 bacteria-treated and control groups at p<0.05.

Additionally, plants treated with the bacteria showed notable increases in antidiabetic activities, with α-amylase inhibition and α-glucosidase inhibition rising by approximately 24.7% and 49.4%, respectively (*p*<0.05). In contrast, there was a modest reduction in the glycemic index, decreasing by about 6.9% (**Figure 7**).

## 4 Discussion

### 4.1 JW0823 could improve growth and tissue chemical composition of *E. sativa*

PGPB can colonize plant roots, improving plant growth (Beneduzi et al., 2012). Our research findings revealed that *E. sativa* plants inoculated with JW0823 showed improved growth compared to non-inoculated plants. In line with our results, previous studies highlighting positive effect of PGPB (e.g., *J eotgalicoccus* sp.) such as enhanced yield and nutrient content (Saleh et al., 2010; Chavoshi et al., 2018; Misra et al., 2019). *J eotgalicoccus* sp. can stimulate plant growth and yield (Gong et al., 2016; Misra et al., 2019; Singh et al., 2019; Li et al., 2022). In this context, *J. huakuii* NBRI 13E has been used in bioinoculant formulations to boost crop yield and quality (Misra et al., 2019). This positive effect of PGBP can be explain by their ability to induce the production of growth hormones (e.g., IAA) (Egamberdiyeva and Höflich, 2004), solubilization of phosphate and mineralization of nutrients (Jeon et al., 2003; Glick, 1995). In this regard, *J. halotolerans* was capable of producing growth-promoting hormones auxin (Mukasheva et al., 2016). IAA is well-documented for their role in enhancing plant growth by promoting cell elongation and division (Mishra et al., 2022).

The growth improvement can also be explained by the bacteria’s ability to enhance nutrient uptake. The potential of bioinoculants as valuable tools in sustainable agriculture, offering benefits through enhanced nutrient uptake. The application of JW0823 enhanced nutritional accumulation in *E. sativa*. Previous studies highlight the role of PGBP in enriching plants with macro- and micronutrients (Yaghoubi Khanghahi et al., 2018; Meena et al., 2017; Ipek, 2019; Kumar et al., 2022; Helaly, 2017) and increasing soil mineralization (Shen et al., 2004; Esitken et al., 2010). For instance, *Jeotgalicoccus* sp. *BAK1* exhibits both growth-promoting and phosphorus-solubilizing properties (Mukasheva et al., 2016). Similar to *J. halotolerans*, other PGBP such as *Pseudomonas* and *Bacilli* are effective microorganisms in the solubilization of phosphate. In this context, Goswami et al. (2016) demonstrated increases in organic acid secretion, including mineral solubilization in soil (Tahjib-Ul-Arif et al., 2021).

Pervious research also demonstrated that PGBP can stabilize CO_2_ levels, regulate stomatal conductance, and boost photosystem II efficiency (Esitken et al., 2010; Orhan et al., 2006; Chen et al., 2016). For example, *J. huakuii* improved maize growth through increasing chlorophyll content (Misra et al., 2019). Applying JW0823 boosts photosynthesis and soluble sugar production, which is essential for synthesizing primary (essential oils, unsaturated fatty acids) and secondary metabolites (polyphenolics) in plants (Misra et al., 2019). Our results also should that inoculated plants had higher protein, carbohydrate, and lipid contents compared to non-inoculated plants. In line with our results, PGBP boosted carbohydrate metabolism in *E. sativa* (Wang et al., 2022b; Ilyas et al., 2020, and Upadhyay and Singh, 2015) and protein and carbohydrate content in bean seeds (Stefan et al., 2013). It is noteworthy that carbohydrates not only support photosystem II but also serve as energy sources for maintain growth (Sami et al., 2016). The observed increase in protein and amino acid levels may be attributed to the ability of PGPB to enhance the uptake of NH_4_ and NO_3_, which are then converted into free amino acids (Johansen et al., 1996; Kumar et al., 2015).

PGPB-induced production of bioactive metabolites contributes to the synthesis of secondary plant metabolites (Zhou et al., 2021). For example, high phenolic and flavonoid contents have been reported in buckwheat plants inoculated with PGBP (Briatia et al., 2018). *J. huakuii* has also been shown to boost maize growth by producing bioactive compounds such as antioxidant phenolics and proline (Misra et al., 2019). When compared to the control, *Jeotgalicoccus* sp. increased the phenol and flavonoid content in cluster beans (Upadhyay and Singh, 2015), suggesting improved antioxidant power (Sarkar et al., 2021). On the other hand, phenolic compounds accelerate the symbiotic relationship between plants and microorganisms (Mandal et al., 2010). They can also facilitate oxygenation reactions, and thus, beneficial bacteria may help inhibit oxidizing enzyme activity (Notununu et al., 2022).

### 4.2 JW0823 improved the biological activity of *E. sativa*

The higher bioactive metabolite content in *E. sativa* boosts its biological value, as it is rich in essential amino acids and polyunsaturated fatty acids, which are crucial nutrients (De Carvalho and Caramujo, 2018). JW0823 has been observed to significantly enhance the biological activity of plants and facilitate the exchange of nutrients, leading to the accumulation of bioactive metabolites. For instance, increased antioxidant content in *E. sativa* was accompanied by substantial enhancements in antioxidant activities (FRAP). *E. sativa* been reported to exhibit high total antioxidant capacities, as well as significant scavenging activity against ROS (Sarikurkcu et al., 2017). In agreement, plants exhibited high antioxidant capacities when treated with PGPB (Chen et al., 2014). Khoobchandani et al. (2010) also investigated the antimicrobial potential of various solvent extracts of *E. sativa* and seed oil against gram-positive (*Staphylococcus aureus* ATCC 6538 and *B. subtilis* MTCC 441) and gram-negative (*E. coli* ATCC 14169, *P. aeruginosa* MTCC 424, and *S. fexneri* MTCC 1457) bacteria that are resistant to antibiotics. They have also been connected to antifungal (Manici et al., 1997) and antinematode (Lazzeri et al., 1993) properties (Warton et al., 2001). However, the research on their antiseptic qualities is limited (Abdou et al., 1972; Hashem and Saleh, 1999). Overall, anti-diabetic, antibacterial and antioxidant properties of *E. sativa*, coupled with its nutritional value, make it a beneficial component of a balanced diet aimed at promoting wellness and preventing chronic diseases.

## 5 Conclusions

Inoculation with JW0823 notably boosted the growth and nutritional quality of *E. sativa*, enhancing its proximate composition, vitamin content, and bioactive metabolites. The treated plants also exhibited increased antioxidant, antidiabetic, and antimicrobial activities. Building on these promising results, future research should explore the broader application of JW0823 in various crop species and agricultural settings to confirm its versatility and efficacy. Further studies could also investigate the long-term effects of JW0823 on plant health and soil sustainability. Additionally, understanding the underlying mechanisms by which JW0823 enhances metabolite production and stress resilience could lead to optimized bioinoculant formulations and targeted application strategies. assessing the practical benefits and scalability of this approach in sustainable agriculture.

## Data Availability

The raw data supporting the conclusions of this article will be made available by the authors, without undue reservation.
